# The C-inefficiency of the EU-VAT and what can be done about it

**DOI:** 10.1007/s10797-021-09683-0

**Published:** 2021-07-29

**Authors:** Sijbren Cnossen

**Affiliations:** 1 CPB Netherlands Bureau for Economic Policy Analysis, Tapijtweg , 2597 KG The Hague, The Netherlands; 2 University of Pretoria, Pretoria, South Africa

**Keywords:** VAT, European Union, Exemptions, Tax reform, C-efficiency, C-inefficiency, H25, H70

## Abstract

It is widely agreed that in countries without major constraints on administrative capacity, a value-added tax (VAT) should tax all goods and services at a uniform rate. In these countries, VAT’s C-efficiency, that is, actual revenue over potential revenue, should be one if compliance is perfect. Under this approach, VAT’s C-inefficiency—the aggregate of the policy gap (exemptions, reduced rates, thresholds) and the compliance gap (revenue shortfalls due to laps in compliance and implementation)—is treated as a residual. This contribution shows that calculating VAT’s C-inefficiency independently of its C-efficiency produces a more telling benchmark, particularly of the policy gap. This is illustrated by an analysis of the revenues of the Dutch VAT, which, given the common VAT directive, should be representative of the VATs in other European Union Member States. The large policy gap, hovering around 0.50, forms the background for exploring three options to improve VAT’s performance: reforming the common directive, ceding VAT design to Member States, and introducing a common modern VAT which can be piggybacked by Member States.

## Introduction

The harmonized value-added tax (VAT) of the European Union (EU) is anything but a modern consumption tax that taxes all goods and services at a uniform rate. In the 1960s when the VAT was adopted, the role and design of an efficient revenue-raising broad-based consumption tax were not fully understood.^1^ To mimic the perceived burden distribution of the previous turnover taxes, multiple exemptions and exclusions^2^ were built into the VAT regime for public policy reasons (healthcare, education, social, cultural, postal and broadcasting services), because it was believed that the tax credit method could not be applied (leasing and letting of immovable property, financial services and insurance, betting and gambling), because it was politically considered not feasible to include agriculture in the base, or simply because it did not seem to make sense to have VAT charged to and paid for on an in-and-out basis by the same taxable entity (governments). The EU’s Common VAT Directive (2006), consolidating the earlier Sixth VAT Directive (1977) and some subsequent amendments, institutionalized these features. Further, various reduced rates were introduced purportedly to mitigate the VAT’s impact on lower-income groups, and a compensation scheme was adopted to keep agriculture outside the VAT’s ambit.

In the meantime, it has become widely recognized that in countries without major constraints on administrative capacity, the VAT should only be used to raise revenue, predictably and efficiently, leaving it to the income tax and social benefit schemes to address distributional concerns and to excise duties and subsidies to deal with negative and positive externalities. In these countries, VAT should tax all goods and services at a uniform rate. This distorts the efficient allocation of resources brought about by the market as little as possible, as relative prices do not change. With reference to the production-efficiency theorem of Diamond and Mirrlees (1971), Crawford et al. (2010) argue that optimality considerations regarding base and rate differentiation do not fundamentally alter this guiding principle for VAT design.^3^ It follows that exemptions should be limited to those strictly necessary on administrative grounds and that reduced rates should not be contemplated. In this respect, the EU-VAT leaves much to be desired. The issues are important because it has been shown that base-broadening and rate unification offset by a reduction in the standard rate fosters economic growth (Acosta-Ormaechea and Morozumi 2019) – more than would an increase in the standard rate in combination with a reduction in the income taxes.

As a large body of theoretical and empirical literature (e.g., De la Feria & Krever, 2013; Ebrill et al, 2001) testifies, exemptions and multiple rates generate welfare costs that should be avoided. Apart from tax-on-tax effects (called cascading, occurring at intermediate stages of production and distribution), exemptions distort input choices, give rise to a self-supply bias, distort competition, cause delineation issues, complicate input VAT allocation over exempt and taxable supplies, favor imports, discriminate against exports, and induce ‘exemption creep.’ Similarly, differentiated rates distort consumer and producer choices.

In an early but still relevant study, Copenhagen Economics (2013) calculated the efficiency gain that can be reaped if various exempt supplies are taxed (with credit for the VAT on intermediate goods and fixed assets). It analyzed the effects of six core services, that is, cultural activities, education, health care, garbage collection, postal services, and radio and television broadcasts, which, jointly, contributed 14.2% of GDP in the EU-27 (excluding Croatia) in 2010. The efficiency gain of fully taxing these services, which is what modern VATs do, would be 0.34% of GDP. If governments would be included, the gain might well approach 0.5% of GDP. By eliminating the self-supply bias, Copenhagen Economics notes, full taxation should have a significant positive economic effect, particularly on medium-sized and small businesses that account for 60% of business services in the affected sectors. Not surprisingly, Copenhagen Economics (2013, p. 12) concludes: ‘we recommend to look towards a full taxation solution.’

The conclusion is inescapable: the EU-VATs are not the efficient revenue-raising instrument that they can be. Current VATs violate basic neutrality norms and thus the single market’s equal playing field principle. The degree to which this is the case is generally measured by the VAT’s C-efficiency,^4^ that is, actual revenue raised over potential revenue found by applying the standard rate to total consumption expenditures of households, non-profit entities and governments. This means that the C-efficiency should be 1 (one) for a uniform-rate VAT on all goods and services, assuming full compliance. The difference between 1 and the actual C-efficiency comprises the VAT that is not levied on account of the policy gap (exemptions, reduced rates, thresholds) and the compliance gap (the difference between what should be collected given existing legislation and what is actually collected). This may be referred to as VAT’s C-inefficiency.^5^ The policy gap dominates the compliance gap in the EU.

The problem with this approach is that C-inefficiency emerges as a residual: 1 – C-efficiency. This says nothing about its component parts: which goods and services are taxed at reduced rates or exempt but subject to distorting input taxes. Much appears to be said therefore for calculating C-inefficiency independently of C-efficiency. This can be done by applying the standard rate to consumer expenditures that are exempt and subtracting any VAT that is paid on inputs (fixed assets and intermediate inputs), and aggregating the individual items. Accordingly, C-inefficiency focuses on specific areas in which VAT is underperforming, and the results can thus be used to establish priorities of reform.^6^ By contrast, C-efficiency merely connotes that there may be room for improving VAT performance, but is silent on details.

Although the large VAT policy gap is a concern in the EU, original sin lingers on. The European Commission concentrates its research on narrowing the compliance gap.^7^ It calls this the VAT gap (European Commission, 2020), ignoring the much larger policy gap, because that means that it has to come to terms with the shortcomings of the common directive. In the same vein, it continues to hammer on the anvil of the introduction of a ‘definitive regime:’ applying the VAT to intra-EU exports with credit for this tax by importers in other Member States along with the transfer of exporters’ VAT payments by exporting Member States to importing Member States, thus maintaining the destination principle.^8^

To put VAT policy back on the agenda, this contribution renews the call for basic VAT reform. It illustrates the distortions and complexities of exemptions and lower-than-standard rates by analyzing the C-inefficiency of the Dutch VAT, which, in view of the mandatory exemptions of the common directive, should be representative of the VATs in most other Member States. This is followed by a proposal to unify differentiated rate structures. Subsequently, ways are explored to remedy the VAT base gaps by reforming the common directive, assigning VAT base design to the Member States, or by adopting a modern, common EU-wide VAT that can be piggybacked by the Member States.

## The C-inefficiency of the Dutch VAT

Based on the Appendix, Table 1 presents an analysis of the policy and compliance gaps of the Dutch VAT. Column 1 shows a breakdown of potential revenues if a modern VAT was imposed by applying the standard rate to all goods and services.^9^ Note that with respect to the VAT base, government salaries for education, medical and social care, and cultural activities have been allocated to the respective consumption categories. Ultimately, consumers benefit from and pay for these services (also Keen, 2013). Column 2 presents a breakdown of actual collections, assuming full compliance, divided into output-taxed transactions (standard or reduced rate) and input-taxed transactions (exemptions). Next column 3 shows the various policy gaps computed by applying the standard rate to the exemptions listed in the Appendix and reducing the outcomes by the input taxes on related fixed assets and intermediate purchases. Column 4 then indicates the degree of ‘under-taxation’ as a percentage of potential VAT. This then can be taken as an indication of the inefficiency of the reduced rate and the various exemptions.^10^

**Table 1:** The Netherlands: VAT Revenue, Policy and Compliance Gaps in 2017 (in € billion). *Source*: Appendix. Totals may not add because of rounding

	Modern VAT	Current full compliance VAT	Policy gap	
			Amount	Percent
	(1)	(2)	(3)	(4 = 3/1)
*1. Output taxed (dual rate)*	*40.3*	*30.4*	*9.9*	*24*
Standard rate (21%)	23.0	23.0	0.0	90
Reduced rate (9%)	17.3	7.4	9.9	57
Households	16.9	7.2	9.7	57
-foodstuffs, restaurants, nonalcoholic beverages	(10.7)	(4.6)	(6.1)	(57)
-personal care products	(1.2)	(0.5)	(0.7)	(57)
-durable consumer goods	(0.4)	(0.2)	(0.2)	(57)
-furnishings, house and garden	(0.3)	(0.1)	(0.2)	(67)
-hotel accommodation	(1.3)	(0.6)	(0.7)	(57)
-transportation	(0.8)	(0.3)	(0.5)	(58)
-recreation, culture	(0.9)	(0.4)	(0.5)	(56)
-other goods and services	(1.3)	(0.6)	(0.7)	(57)
Non-profit institutions	0.4	0.2	0.2	57
Governments	–			
*2. Input taxed (exemptions)*	*61.0*	*23.8*	*37.3*	*61*
Households	54.0	17.4	36.6	67
-rents and rental values	(14,1)	(7,9)	(6.3)	(44)
-house and garden	(0.3)	(-)	(0.3)	(100)
-transportation	(0.6)	(0.5)	(0.1)	(12)
-financial/business services	(4.7)	(2.9)	(1.8)	(38)
-education, culture, recreation	(7.3)	(2.1)	(5.2)	(72)
-medical and social care	(25.5)	(3.3)	(22.2)	(87)
-other goods and services^a^	(1.5)	(0.8)	(0.7)	(49)
-threshold, agriculture	(pm)		(pm)	
Non-profit institutions	0.4	–	0.4	100.0
Governments	6.6	6.3	0.3	4.0
Total revenue/Policy gap (lower rate, exemptions)	101.3	54.2	47.1	46.5
Actual revenue (CBS, 2019)		49.8		
Compliance gap		4.3	4.3	4.2
C-efficiency (actual revenue over potential revenue)				0.49
C-inefficiency (VAT gaps over potential revenue)				0.51

^a^ Including small amounts of exempt items of foodstuffs, energy, and communication listed in the Appendix

On the basis of the information in the Table, the C-efficiency of the Dutch VAT, the usual performance indicator, can be computed as 0.49.^11^ Alternatively, and preferred here, the VAT’s (lack of) performance, measured by its C-inefficiency, is calculated as the policy gap of €47.1 billion (attributable to the lower rate and the exemptions) plus the compliance gap of €4.3 over total potential VAT of €101.3 billion, that is, 0.51. The C-inefficiency draws attention to the composition of the VAT policy gap (column 3 in the Table), that is, the goods and services or sectors that are undertaxed and the extent to which this is the case. Similarly, it shows the size of the policy gap that can be attributed to the reduced rate.

The exemptions in Table 1 are mandatorily prescribed by the Common VAT Directive (2006), namely in Article 13 (public bodies excluded from VAT), Article 132 (exemptions for specified activities in the public interest), and in Article 135 (exemptions for other activities). In addition, EU Member States can provide a threshold below which small entities, which might have difficulties in complying with the VAT’s obligations, are exempted from VAT.^12^ For similar reasons, the agricultural sector can be left out of the VAT base.^13^

As indicated in Table 1, only 23% of the potential base is subject to the standard rate and 17% to the lower rate. By contrast, the total amount of exempt goods and services that are input-taxed comprises 60% of the potential VAT base, indicating the lack of integrity of the actual VAT. The VAT on the inputs for these goods and services, which cannot be invoiced to customers, constitutes an indeterminate and capricious element in sales prices, which distorts producer and consumer decisions and greatly complicates the application of the VAT. Further, the reduced rate accounts for €10 billion of foregone revenue. Not surprisingly, many newcomers to the VAT tax goods and services much more comprehensively and apply a single rate. New Zealand is the prime example of a country that has the most modern VAT, called Goods and Services Tax (GST), in the world.

The capricious taxation of most goods and services is also apparent from the effective tax rates shown in column 9 of the Appendix, that is, actual VAT receipts as a percentage of the modern VAT base. The standard rate of 21% is only applicable to alcoholic beverages, tobacco, energy, motor fuel, durable consumer goods and communication services. Further, the effective tax rate on foodstuffs, restaurants, nonalcoholic beverages and hotels equals the nominal rate of 9%. As expected, exempt services are taxed at lower effective rates, ranging from 2.8% on medical and social services to 14% on financial and business services.

Looking at the data in the Appendix from another angle: the overall effective tax rate is approximately 11%, one half of the standard rate. In other words, the standard rate of 21% could be lowered to 11% if all goods and services were taxed uniformly at that rate (ignoring in-and-out effects of the VAT on exempt public bodies). Another interesting feature is that the Dutch VAT’s C-inefficiency would be 0.42 if medical and social services, education (not recreation and culture) and governments would be zero-rated, as proposed below, along with appropriate reductions of the modern VAT base. This would eliminate the input choice distortion and the self-supply bias of these sectors (albeit not the competitive distortion).^14^ To sum, the exemptions and lower rate result in a patchwork quilt of effective tax rates–a situation that is difficult to justify in the Netherlands capable of levying a modern VAT and having an advanced social benefit system to compensate lower-income groups for the regressive impact of the VAT.

## Unifying the dual rate structure

With the exception of Denmark, most Member States have differentiated rate schedules. The Appendix (in the column Notes) lists the items subject to the reduced rate in the Netherlands. The reduced rates, especially on foodstuffs, are supposed to mitigate the VAT burden, measured against disposable income,^15^ on lower-income households, although Bettendorf and Cnossen (2015) show that in the Netherlands higher-income groups benefit nearly twice as much in absolute terms from the lower rate on foodstuffs (and cultural activities) than lower-income groups–an odd way of trying to help the poor.

The lower-rate’s application to income-elastic items of consumption, such as restaurants,^16^ hotels, books, periodicals, museums, theaters and horticultural products is difficult to defend on equity grounds. Further, the lower rate on shipping and the exemption for postal services are questionable if other forms of private transportation and communication are fully taxed. Besides, the tax on shipping is creditable by VAT-liable customers.

The economic argument in favor of a uniform rate is that it does not distort consumer and producer choices. Eliminating the reduced rate, therefore, involves a welfare gain, which has been quantified by IFS (2011) for the UK and Belgium at 3.5% and 4.6%, respectively, of total VAT receipts. In an alternative scenario, the elimination of the reduced rate is combined with a reduction of the standard rate by 5%-points, so that VAT yields stay the same. This would result in a welfare gain per household of €1.07 per week in the UK and €0.74 per week in Belgium.

A further argument in favor of a uniform rate is that it contributes to a simpler VAT system with lower administration and compliance costs. In 2008, the Dutch Ministry of Finance (Ministerie van Financiën, 2008) estimated that a uniform VAT rate would reduce compliance costs by some €100 million. Also, the direct administrative costs of the VAT, estimated at €138 million, would go down. Compared with the dual rate structure, a uniform rate is also less sensitive to lobbying activities, while misclassifications, intentional or not, should not occur.

Table 2 clearly implies that the disadvantages of a differentiated rate structure should not be underestimated. Several rate differentials for similar goods and services are hard to justify and must be nearly impossible to monitor. A curious form of gender discrimination is that the services of a stallion are taxed effectively at 12% (by subjecting 3/4^th^ of the consideration to the 9% rate) and the services of a breeding mare at 15% (taxed on a 50/50 basis between the lower and the higher rate).

**Table 2:** The Netherlands: Examples of VAT rate differentiation. *Source*: Belastingdienst, BTW tarieven en vrijstellingen (2021). Available on the internet

Reduced rate (9%)	Standard rate (21%)
Tap, distilled and warm water	Frozen and demineralized water, steam
Grains, rice, barley and oatmeal flour	Pea and bean flour
Cookery course with dinner	Cookery course as knowledge transfer
Dried flowers, bouquets < €7 sold at wholesale	Painted and artificial flowers
Haircut human	Haircut dog
Books, newspapers, coloring book	Writing pads, tear-off calendars, travel and hotel guides
Operas, movie theaters	Peepshows
Piano recital	Counseling piano rehearsal
Admission ticket for lecture	Admission ticket for seminar
Radio/TV presentation of own work	Radio/TV presentation of other people’s work
Condoms	Lubricants
Sauna	Tanning bed
Bicycle repair	Moped repair
Skate shoe repair	Skate sharpening
Roller	Walking stick
Rabbit feed	Guinea pig feed
Fish offal	Fish feed
Flee poison pill for cat administered by owner	Flee poison pill administered by veterinarian
Dressings for horses	(Medical) pet shirt
Stallions (12%)	Breeding mares (15%)
Rearing horses	Training horses
Tent for camping guests	Tent for non-camping guests
Leasing busses	Leasing single bus
Cleaning homes < 2 years old	Cleaning homes > 2 years old

## Modernizing the EU VAT base

The first option to tackle the economic distortions and administrative complexities of the current VAT is modernizing the common directive along the lines of the New Zealand GST. The most important exemptions, discussed below, that would have to be reviewed are: immovable property, financial services, insurance, lotteries and gambling, education, recreation, culture, medical and social services, and governments.^17^

### Immovable property

The Common VAT Directive (2006) exempts all used immovable property, residential and non-residential, subject to a registration option for non-residential property. Newly created property is taxed, the assumption being that the VAT on the purchase price may be considered a proxy for the discounted value of the VAT that should have been levied on the future flow of building services. By contrast, the modern VAT only exempts sales of used residential property, a more comprehensive and therefore less distortionary approach as future increases in value of non-residential property are also included in the base.^18^ Also, a modern VAT does not need a definition of specified non-residential use, such as hotel accommodation, boarding houses, camping facilities, and parking space that are taxable under the EU VAT on the rates charged to customers. These exceptions bring their own definitional and administrative difficulties along. Ideally, the modern approach should be complemented by the taxation of the increase in the value of used residential buildings realized at the time of sale (while refunds should be made if the value has declined), similar to the VAT margin scheme for second-hand goods (Cnossen, 2011). Under a modern VAT, the margin scheme is also applied to land, which shelters land from taxation except on the increase in value (Cnossen, 2019).

### Financial services

The Common VAT Directive (2006) exempts financial services entirely, whether fee- or margin-based. This implies that business-to-consumer (B2C) services tend to be undertaxed and business-to-business (B2B) services overtaxed. By contrast, modern VATs tax fee-based services without further ado, which raises the tax on B2C services and enables business users of these services to credit the tax on fee-based services in full. Admittedly, some fee-based services might be replaced by margin-based services, but disintermediation has made this less likely (Poddar, 2003).

This leaves the distortionary effect of the exemption of margin-based services rendered to taxable businesses, which are not entitled to a credit for the VAT included in their price.^19^ To remedy this defect, consideration should be given to allowing partial formula-based recovery of the input VAT incurred in rendering B2B margin services by financial institutions**.** Australia, New Zealand and Singapore have instituted rules to that effect. New Zealand, for instance, zero-rates supplies of financial services to non-financial businesses if their turnover, measured over a 12-month period, consists of at least three-quarters of taxable supplies.

Alternatively, financial services could be taxed under an addition-method VAT on the sum of payroll and business cash flow. Denmark and Israel do so, although Denmark does not include business cash flow in the base and Israel taxes profits instead of business cash flow. Iceland and Norway have similar taxes on financial institutions. Although these approaches result in a fuller taxation of B2C services, they do not permit the passing on of input VAT on a transaction-by-transaction basis and, hence, increase the distortion of the VAT with respect to B2B financial services.^20^ Hence, they are not recommended in solving the policy gap for financial services.

The ingenious cash-flow method, developed by Poddar and English (1997) and elaborated on by Poddar (2003), would be another alternative to apply the VAT properly to financial services. It essentially bypasses the problem of determining the pure rate of interest, yet fully taxes consumer use of financial services and not business use. The method has been tested in an operational sense with ten large financial institutions in six EU Member States. Apart from the fear of the unknown, complexity and compliance costs have been the central source of objection to the scheme (Kerrigan, 2010). As a result, it has not been implemented anywhere.

### Property and casualty insurance

Insurance services are exempted from VAT in the EU, because at the level of the policyholder the taxable intermediation charge cannot be separated from the non-taxable capital transfer to the common pool from which indemnity and contingency payments are made. By contrast, modern VATs avoid the problem of determining taxable value added on the basis of individual policies by shifting taxation to the level of the insurance company, where value added is the difference between premium receipts, on the one hand, and indemnity payouts and taxable purchases, on the other. This difference is taxed by collecting VAT on insurance premiums (and permitting a credit for the VAT on the taxed inputs of insurance companies), and imputing a tax credit to indemnity payments (to be offset against the VAT on premiums).^21^ The VAT on insurance premiums would be creditable by clients liable to VAT, who should be obliged to include any imputed tax credit received (along with indemnity payments) in their VAT return.

While property and casualty insurance can thus be taxed, health and life insurance should probably remain exempt (or be zero rated). Both forms of insurance are mostly taken out by individuals; hence, cascading effects are unlikely to occur (and thus, taxation on the basis of the sum of payroll and business cash flow could be considered an alternative). For VAT purposes, health insurance would then be treated in the same way as other health services if these were to remain exempt, while life insurance—a long-term savings vehicle akin to pensions—would be exempted along with other financial margin-based B2C services.

### Lotteries and gambling

EU practice is to exempt games of chance from VAT. In contrast, countries with a modern VAT tax most games of chance. Lotteries can be taxed on ticket sales (output), while a reverse charge can be imputed to payouts (inputs), similar to the modern VAT treatment of property and casualty insurance. A credit for VAT on other inputs should be allowed against the tax liability (for a fuller treatment, see Schenk, 2010). Casinos can be included in the VAT base under the margin method, where the margin is the difference between the sales of tokens and chips, on the one hand, and payouts, on the other. Both approaches yield the same result. VAT should be applied to transactions involving games of chance, regardless of whether or not lotteries and gambling are subject to externality-correcting excises.

### Healthcare, education, social and cultural services

Healthcare, education, and various social services—called activities in the public interest—are often considered ‘merit goods’ whose consumption should not be constrained by the imposition of VAT. But even if the merit-good argument would be acknowledged, full taxation of the output (including subsidies, if any) of hospitals, schools, universities and various forms of social assistance in combination with increased subsidies could leave the total net amount of the (VAT-inclusive) charge for the services unaffected without distorting the exempt entities’ input choices and outsourcing activities and without discriminating against similar taxable services provided by the private sector.

Accordingly, under New Zealand’s GST, supplies by health organizations, educational institutions (except elementary schools), cultural organizations, social assistance agencies, child welfare groups, postal services, and public broadcasting companies, all exempt in the EU, are taxable. According to Aujean et al. (1999), this has ‘permitted there [i.e., in New Zealand] a dramatic simplification of VAT rules as they apply to public bodies.’ The effect on revenue, moreover, would be nil to the extent that the charge for the activities as well as their financing are determined by public bodies. Much the same effect is attained in Canada, which refunds the GST on the inputs of the MUSH sector: Municipalities (up to 100%), universities (67%), schools (68%) and hospitals (83%), percentages that have increased over time and that are moving toward full refunds (Gendron, 2013). Note, however, that the refunds may be aggravating the competitive distortion with regards to the private sector.

### Governments

Governments—central, regional and local—and other public bodies are considered out-of-scope of the EU-VAT, subject to some highly contentious conditions (as evidenced by a vast body of jurisprudence reviewed by Henkow (2013)), that they should be governed by public law, that they should be operated under public authority, and that their activities should not involve significant distortions of competition. ‘Out-of-scope’ expresses the view that governments should be viewed as final consumers, i.e., not be subject to tax and therefore not entitled to credit for the tax on inputs. Incidence theory, however, holds that only people, not institutions, bear taxes; their disposable income is reduced. Accordingly, services provided by governments to their citizens/consumers should be taxed in full, if feasible, just like goods and services produced by the private sector. Exceptions should only be made for pure public goods (defense, public administration, law and order) for which it is not possible to set a ‘price,’ and for the redistribution of income and wealth which does not constitute consumption (see the influential paper by Aujean et al., 1999).

Although in terms of revenue, the taxation of many goods and services provided by public bodies would largely be in the nature of a pay-out and claw-back arrangement, the VAT on the inputs of exempt bodies causes the same non-neutralities and complexities as other exemptions: distortion of input choice, self-supply bias, unfair competition, and tax avoidance. Summing up the situation, Aujean (2010, p. 514), an astute observer and longtime participant in the debate on EU-VAT, has labeled the VAT treatment of public bodies ‘complex, inefficient, costly, and legally uncertain’.^22^ Apart from eliminating most distortions, delineation issues regarding taxable versus non-taxable activities would become redundant. Although public bodies do not pursue profit maximization per se, cost minimization should be their aim and this goal is promoted by applying the VAT as widely as possible.

The main issue that arises in taxing public goods and services is that many tend to be made for nil, nominal, or break-even consideration and that they are usually financed through a variety of means which include charges, user fees, taxes, subsidies, grants, budgetary allocations and funds from borrowing, often without a direct link to the supplies. Under modern VATs, all of these means of financing, regardless of their form, are included in the VAT base. Full inclusion prevents tax avoidance.^23^ The full taxation of public bodies, as is done in New Zealand, would, of course, require adjustments on the expenditure side of the budget.

An alternative, found in Australia and Canada, would be to zero-rate (refund the input tax) municipalities, provincial governments and the central government without further ado (Gendron, 2013). This would also eliminate most distortions and complexities, except for the competitive advantage governments would have if goods and services can also be provided by the private sector which would be subject to VAT.^24^ Copenhagen Economics (2013) estimates that the zero-rating of the services included in its study, as opposed to full taxation, would increase welfare by 0.02% of GDP. Clearly, the situation calls for deeper reforms, preferably full taxation or close to comprehensive refund schemes, as in Australia and Canada.

## Assigning VAT design to member states

The difficulty of making changes to the common directive is that they require the unanimous consent of all Member States, an important obstacle to reform. But if a consensus on the adoption of improvements to the common directive cannot be reached, perhaps Member States themselves should be allowed to modernize their VATs. Arguably, a modern VAT, which affects the principal neutrality requirement of the single market less than the current VAT, can hardly be considered to infringe on the principles of free trade and free competition, that is, violate the EU Treaty.^25^ To ensure that this is the case, perhaps VAT reforms of Member States should be subject to approval by the Brussels-based VAT Committee.

In going down this road, it seems useful to dwell briefly on the rationale of the EU-VAT. Early on, the EU’s founding fathers realized that the principle of free trade and free competition between and, by extension, within Member States, required that taxes on goods and services should be fully and accurately rebated at export, equivalently imposed at import, and washed out between taxable businesses within Member States. To achieve this, the cumulative turnover taxes, levied in five of the six founding Member States, were replaced by the VAT, which, in principle, embodies these neutrality features. While correct, it was also thought, incorrectly, that harmonizing the VAT base would help achieve this objective. This bypassed the a priori issue which base should be harmonized. Instead of taking a de novo approach, the founding fathers opted for some average of existing VAT bases. This meant that the inherent distortions and complexities of the exemptions were institutionalized, notably in the VAT Sixth Directive (1977), which, following some minor amendments, was succeeded by the VAT Common Directive (2006). To top it all off, accepting the common directive became a *conditio sine qua non* for joining the EU.

On this basis of this reasoning, it appears that the guiding criterion for the VAT Committee in evaluating VAT reforms of individual Member States should be: do reforms enacted by Member States in redesigning the base of their VATs violate the principle that intra-EU imports should not be taxed higher than similar domestic products, and that exports should not be subsidized by refunding more tax than levied in previous stages of production and distribution? If not, presumably reforms should be allowed to proceed.

## Introducing an EU-wide VAT

Before considering the last option, that is, an EU-wide VAT, an answer should be sought to Musgrave’s famous question: “Who Should, Where and What?” (Musgrave, 1983). In other words, what does tax assignment theory say about the allocation of taxing rights within a single market or, in institutional terms, a (con)federation? Currently, the EU does not have explicit power to tax, although it can impose import duties and administers a common emissions scheme, which is similar to a tax. In addition, the European Council, in launching the Next Generation EU recovery program (NGEU) in response to the COVID-19 crisis, has requested the European Commission to make proposals for new own resources that can be used for early repayment of NGEU borrowing. Potential candidates for these resources are a carbon levy on imports, a charge on nonrecycled plastic, a digital tax, a tax on the profits of EU-wide multinational companies, and a financial transactions tax (Fuest & Pisani-Perry, 2020). These are all levies that require EU-wide decision making in design. The mandate also resembles the contours of a federal tax covenant.^26^

Musgrave (1983) suggests that regional governments in a federal setup should have a broad destination-based sales tax, be it a VAT or a retail sales tax, in view of its relatively low interjurisdictional mobility which limits the possibility of “tax burden export.” Further advantages are that the base of such a tax is fairly evenly distributed across the federation, its yield is relatively stable and, importantly, sizable (Ter-Minassian, 1997). Accordingly, finance can follow function (Bird, 2010), as the Member States have important expenditure obligations regarding health, education, medical and social care. In this view, the VATs, like the retail sales taxes in the United States (McLure, 2000) should remain the “fiscal lifeblood” of the Member States. Subnational fiscal autonomy, in particular the authority to levy taxes, can lead to significant improvements in social welfare (Garcia-Mila et al., 2018).^27^

This still means, of course, that spillover effects of bad VATs should be avoided. Moreover, it would not preclude the introduction of a low-rate modern EU-wide VAT, which, in Musgrave’s philosophy, might be desirable for stabilization purposes. Such a VAT could be a blueprint for the Member State VATs and might be instrumental in closing the compliance gaps of the Member States, because it would be in a pivotal position to audit intra-EU transactions and, stretching this point further, in providing information for and auditing other taxes and levies with EU-wide implications.

The model for a dual VAT can be found in Canada which has a federal GST piggybacked by five out of ten Provinces in the form of Harmonized Sales Taxes (HSTs for short), that is, separate HST-rates on top of the Federal VAT rate, applied to more or less the same base.^28^ Interestingly, HST-rates differ one from another and to some extent, so do the bases. Noteworthy also is that Quebec administers the federal GST pertaining to the province along with its own VAT. To illustrate further how much diversity is possible with proper coordination, three Canadian Provinces still retain their own retail sales taxes, and one, oil-rich Alberta, does not levy a broad-based sales tax at all. Reportedly, the Federal/Provincial GST regime, administered by Revenue Canada, works well. Provincial revenue shares are determined on the basis of ‘final’ consumption.

## Summary and conclusions

The role and proper design of the VAT were not fully understood at the time the EU-VAT was introduced. Instead of designing it as an efficient revenue-raising instrument, multiple exemptions and differentiated rate schedules were introduced to try and mimic the distributional pattern of the old turnover taxes. The deviations from what is widely regarded as a modern VAT were institutionalized in the form of a common directive. As a result, the EU-VAT tends to become an anachronism (Cnossen, 2003), as illustrated by an analysis of the C-inefficiency, calculated independently, of the Dutch version. The contribution shows that the VAT policy gap is large, generating substantial welfare costs.

Three options are considered to modernize the EU-VAT. Firstly, VAT base broadening would involve the mandatory inclusion of non-residential property in the VAT base, the taxation of fee-based financial services and the recovery of input taxes on B2B services, the taxation of property and casualty insurance, lotteries and gambling, postal services and public broadcasting. It would also involve the taxation of governments, healthcare, education, social and cultural services along with an increase in subsidies. Alternatively, and perhaps more appealing, the services provided by these activities could be zero-rated along with adjustments of government budget expenditures. This would eliminate input choice distortions and self-supply biases, albeit it might worsen competitive distortions. Further, the differentiated rate schedules could be unified and the flat-rate compensation schemes for farmers abolished.

Secondly, if agreement on VAT reform cannot be reached, consideration could be given to ceding VAT base design to the Member States, subject to the condition that intra-EU trade should not be affected to be ascertained by the EU VAT Committee. Member States would then be able to adopt a modern VAT, which taxes all goods and services at a uniform rate.

Thirdly, a low-rate modern VAT could be imposed on an EU-wide basis, which would greatly improve the ability to audit intra-EU transactions and which would be helpful in ascertaining the compliance with other EU-wide taxes and levies. Such a tax could be piggybacked by individual Member States along the Canadian example. Rate differentiation between, not within, Member States should be possible, as well as differences in VAT base design although not advisable.

All these proposals have in common that the VAT bases and rate structures should be modernized. It would seem worthy of consideration, therefore, if the European Commission were to draw up a model of a modern common VAT along with an explanatory memorandum as a focus for discussion in the EU.

## Appendix

**Table:**

**Consumption expenditures** (incl. in kind social benefits and wages; incl non-residents but not residents abroad)	**Base modern VAT** (3 + 4 + 5)	**Base actual VAT**						**Yield and rate**	
		**Output taxed**		**Input taxed**				**Tax**	**Effective tax rate (%)**
		**Standard rate**	**Reduced rate**	**Exempt**	**Fixed assets** (standard)	**Intermediate use**			
						**Standard**	**Reduced**		
**(1)**	**(2 = 3 + 4 + 5)**	**(3)**	**(4)**	**(5)**	**(6)**	**(7a)**	**(7b)**	**(8)**	**(9 = 8/2)**
**A. Households**	**445,406**	**107,595**	**80,551**	**257,260**	**37,701**	**40,349**	**11,470**	**47,267**	**10.6**
***1. Food, soft drinks***	***52,218***	***1,295***	***50.911***	***12***				***4,854***	***9.3***
Foodstuffs	34,733	1,295^a^	33,426	12				3,280	9.4
Restaurant services	14,630^b^		14,630					1,317	9.0
Water	1,066		1,066					96	9.0
Nonalcoholic beverages	1,789		1,789					161	9.0
***2. Other consumer goods***	***27.633***	***21,687***	***5,692***	***254***				***5,066***	***18.3***
Energy	6,570	6,316		254				1,326	20.2
dPersonal care products	9,504	3,812	5,692^c^					1,313	13.8
Alcoholic beverages	7,523	7,523						1,580	21.0
Tobacco	4,036	4,036						848	21.0
***3. Durable consumer goods***	***29,416***	***27,491***	***1,925***^d^					***5,946***	***20.2***
***4. Housing***	***91,542***	***15,189***	***7,734***	***68,619***	***25,651***	***10,850***	***2,152***	***11,745***	***12.8***
Rents and rental values	67,638	341	82	67,215	25,651^e^	10,850^e^	2,152^e^	7,938	11,7
Home furnishings, decorations	11,897	11,834	63^f^					2,491	20.9
House and garden	5,825	3,014	1,407^ g^	1,404				760	13.0
Hotel accommodation	6,182		6,182					556	9.0
***5. Mobility***	***31,004***	***23,975***	***3,944***	***3,085***	***1,253***	***1,838***	***26***	***6.041***	***19.5***
Vehicles	7,992	7,984	8^ h^					1,677	21.0
Motor fuel	9,296	9,296						1,952	21.0
Transportation	7,358	723	3,936^i^	2,699^j^	1,238	1,104	6	998	13.6
Communication	6,358	5,972		386	15	734	20	1,413	22 0.2
***6.Financial/business services***	***25,508***	***3,009***		***22,499***	***4,076***	***9,446***	***967***	***3,559***	***14.0***
***7.Education, culture***	***40,095***	***1,245***	***4,168***	***34,682***	***2,438***	***6,986***	***922***	***2,699***	***6.7***
Education	24,575^ k^			24,575^ k^	2,246	5,592	539	1,694	6.9
Recreation	9,546	1,108	2,197	6,241^ l^				430	4.5
Culture	5,974^ m^	137	1,971^n^	3,866^ m^	192	1,394	383	574	9.6
***8.Medical and social services***	***122,349***	***827***		***121,522***	***4,193***	***8,540***	***7,068***	***3,484***	***2.8***
Medical services	32,236°	827^p^		31,409^o^				174	0.5
Social care	27,469			27,469				-	-
Medical and social care	62,644^q^			62,644^q^	4,193	8,540	7,068	3,310	5.3
***9.Other goods and services***	***25,641***	***12,877***	***6,177***^r^	***6,587***	***90***	***2,689***	***335***	***3,874***	***15.1***
**B. Non-profit institutions**	**5,661**	**1,968**	**1,737**	**1,956**				**570**	**10.1**
**C. Governments**	**31,470**^ s^			**31,470**^ s^	**12,236**	**17,451**	**1,222**	**6,344**	**20.2**
**Grand totals**	**482,537**	**109,563**	**82,288**	**290,686**	**49,937**	**57,800**	**12,692**	**54,182**	**11.2**

The Netherlands: Composition and Tax Yield of the VAT in 2017 (mln euro). Source: Central Bureau of Statistics, Expenditure categories (excluding VAT) by VAT rates 2017. The base of the modern VAT for the exempt sectors: education, medical and social care, recreation and culture is assumed to consist of the remuneration of (government) employees found in the national accounts (CBS, 2019) in addition to private consumption expenditures on these categories. The column Exempt (5) does not include exempt fixed assets and intermediate use goods and services. Totals may not add because of rounding.

^a^animal feed.

^b^not including alcoholic beverages.

^c^dressings, nonprescription medicines, sun cream, toothpaste.

^d^books, medical instruments and apparatus.

^e^exploitation and trade in real estate.

^f^art objects.

^g^ maintenance services.

^h^walkers.

^i^ public transportation, shipping taxis.

^j^postal services, ambulances.

^k^salaries.

^l^sports, entertainment parks, zoos, a.o.

^m^incl. salaries of € 3,866 mln.

^n^ museums, theaters. a.o.;

^o^ incl. salaries of € 24,399 mln.

^p^paramedical care.

^q^ salaries.

^r^incl. pets, preservatives, periodicals, horticultural products.

^s^salaries.

## Footnotes

1 For a brief history of the introduction of the common VAT in the EU, see De la Feria and Krever (2013), and James (2015).

2 Both under exemptions and exclusions, no tax is charged on output and no credit is provided for the tax on inputs. The only difference between exemptions and exclusions is that exempt entities can opt for registration and payment of VAT, and thus pass their tax on inputs on to their customers. By contrast, excluded entities are always stuck with the tax on inputs. In the following, exemptions include exclusions, unless the context indicates otherwise.

3 Further, Keen (2013, p. 431) points out that “[u]ltimately, the use of uniform taxation as a reference point in evaluating VAT systems reflects the pragmatic judgment that the practical case for a single rate [and a broad base] is so strong as to make this an important benchmark. …. The case is especially persuasive in higher-income countries, a central lesson of the literature being that uniform taxation is more likely to be optimal the more sophisticated the range of other instruments by which equity objectives can be pursued”.

4 See Keen (2013) for the best discussion of the pros and cons of C-efficiency, also called VAT Revenue Ratio (VRR) by the European Commission and the OECD.

5 For a summation of the factors that influence the C-(in)efficiency, see OECD (2020).

6 It should be emphasized that the calculation of C-inefficiency, like the calculation of C-efficiency, is an accounting exercise, useful in identifying the composition of the VAT gap but ignoring behavioral effects.

7 According to the European Commission (2020), the EU Member States jointly lost an estimated €140 billion in VAT revenues in 2018 due to evasion and avoidance. It expected that the revenue loss would rise to of €164 billion in 2020 due to the effects of the coronavirus pandemic on the economy.

8 This definitive regime was originally proposed by Cnossen (1983) and subsequently adopted by the European Commission (1995, 1996). In a subsequent publication Cnossen (2010) argued for a continuation of the deferred payment system (charging VAT on imports to the first inland recipient of intra-EU imports) along with cross-border VAT audits.

9 The standard rate of 21% would be lower of course if the analysis was done on an equal yield basis.

10 As Keen (2013) notes, in welfare terms not much can be read into changes in C-efficiency nor, for that matter, in C-inefficiency.

11 The OECD (2020, p. 97) computed the C-efficiency of the Dutch VAT (excluding governments) at 0.52 in 2017. This was lower than the OECD’s unweighted average of 0.56, and also lower than the average of the other EU Member States (0.52).

12 In 2017, the Dutch VAT did not have a registration threshold. Instead, net VAT-liabilities of small businesses of €1.345 or less in any year did not have to be paid. The small VAT gap on account of this ‘exemption’ has been ignored. From 2020 onwards, the small-business exemption has been simplified and enlarged by introducing a general threshold of €20.000.

13 Before 2018, Dutch farmers were not obliged to register for VAT (although they could opt to do so), while their VAT on inputs was presumptively washed out through a flat-rate compensation scheme providing purchasers of agricultural products a tax credit for the estimated input VAT incurred by farmers. As pointed out by Cnossen (2018), this is an arbitrary form of input relief, out of date and in conflict with a modern VAT. Subsequently, the Dutch government abolished the scheme and applied the normal VAT to farmers. For the calculation of the policy gap, it is assumed that the farmers’ compensation scheme breaks even.

14 I would also lower the tax/GDP ratio by 1.6%, an optical side-effect that some parties might welcome.

15 IFS (2011) and in its wake Thomas (2020) argue that measuring VAT burdens against household expenditure is a more meaningful estimate of the distributional impact of the VAT as it eliminates the distorting impact of savings patterns. On this basis, these studies conclude that the VAT’s burden distribution is broadly proportional and, in some instances, even progressive.

16 Admittedly, an important argument in favor of aligning the taxation of restaurants with foodstuffs is that it prevents tax avoidance.

17 For a similar comprehensive VAT base discussion on which this section draws, see chapter 18 in Cnossen (2019)

18 Incidentally, the EU’s exemption approach is equivalent to the modern tax approach, if, under the former, all owners of non-residential buildings would opt for registration and payment of VAT, but this is not likely to happen. For a full treatment of the exemption and tax approaches, reference is made to Cnossen (2011).

19 Margin-based services are difficult to tax under the tax credit method VAT, because the value of the intermediation charge (which should be taxed) is embedded in interest rates, returns or rewards, which (along with the principal) should not be taxed, because they are not consumption but income. Because the value of the intermediation charge is not known, VAT cannot be charged on it and passed on to clients on a transaction-by-transaction basis, necessary if VAT-liable customers are to credit the tax against their VAT on output (see the leading paper by Poddar, 2003).

20 That is also the case with the Financial Activities Tax (FAT) proposed by the IMF (2010). Similar objections can be raised against the Financial Transactions Tax (FTT) proposed by the European Commission (2011) and found in some Member States. An FTT, moreover, causes cumulative effects that inefficiently stimulate business concentration. For a comparative analysis, see Lockwood (forthcoming).

21 Correct treatment would also imply that insurers’ investment returns, which are eligible for a reverse credit if paid out, should be taxed, although this is not done anywhere. For a review and evaluation of the EU situation and a detailed reform proposal along the lines sketched here, see Cnossen (2013).

22 In the same vein, another able tax lawyer, De la Feria (2009), has documented the highly complex web of conditions, derogations, movements into and out-of-scope of VAT, and transitions between ‘exempt’ situations, ‘taxable’ situations, and ‘non-taxable because out-of-scope situations.’ She has attempted to lay this down in a flow chart, which shows that it is almost impossible to establish and apply a single consistent VAT treatment to all public sector bodies. De la Feria opines that the Common VAT Directive is becoming more out of step with economic realities as time passes. In her opinion, moreover, the EU treatment entails very significant compliance and administrative costs, as well as frequent and costly litigation.

23 As an example, Copenhagen Economics (2013) notes that, if taxed, public bodies can avoid the VAT under present arrangements by transforming product subsidies (taxable under the Common VAT Directive) into budget subsidies (not taxable). Budget subsidies, therefore, should also be included in the taxable base, which is what modern VATs do.

24 Input VAT refunds are also provided under the VAT compensation schemes in various EU Member States, but Wassenaar and Gradus (2004) conclude that their haphazard application hardly solves the self-supply bias and the investment disincentive.

25 Articles 110 to 113 of the Treaty on the Functioning of the European Union (TFEU), specify that the legislation on indirect taxation has to be harmonized in order to create the conditions for the proper functioning of the internal market and to avoid the distortion of competition.

26 The principal difference between a confederation, more or less the present situation in the EU, and a federation appears to be that the latter form of government has independent power to tax.

27 I owe the thoughts in this paragraph to Bird (2008).

28 For detailed write-ups of the Canadian GST regime, see Smart and Bird (2014) and Bird (2013).
